# Corneal Wound Healing Is Compromised by Immunoproteasome Deficiency

**DOI:** 10.1371/journal.pone.0054347

**Published:** 2013-01-24

**Authors:** Deborah A. Ferrington, Heidi Roehrich, Angela A. Chang, Craig W. Huang, Marcela Maldonado, Wendy Bratten, Abrar A. Rageh, Neal D. Heuss, Dale S. Gregerson, Elizabeth F. Nelson, Ching Yuan

**Affiliations:** Department of Ophthalmology and Visual Neurosciences, University of Minnesota, Minneapolis, Minnesota, United States of America; University of Missouri-Columbia, United States of America

## Abstract

Recent studies have revealed roles for immunoproteasome in regulating cell processes essential for maintaining homeostasis and in responding to stress and injury. The current study investigates how the absence of immunoproteasome affects the corneal epithelium under normal and stressed conditions by comparing corneas from wildtype (WT) mice and those deficient in two immunoproteasome catalytic subunits (*lmp7* ^−/−^/*mecl-1* ^−/−^, L7M1). Immunoproteasome expression was confirmed in WT epithelial cells and in cells of the immune system that were present in the cornea. More apoptotic cells were found in both corneal explant cultures and uninjured corneas of L7M1 compared to WT mice. Following mechanical debridement, L7M1 corneas displayed delayed wound healing, including delayed re-epithelialization and re-establishment of the epithelial barrier, as well as altered inflammatory cytokine production compared to WT mice. These results suggest that immunoproteasome plays an important role in corneal homeostasis and wound healing.

## Introduction

The proteasome is a proteolytic complex that plays a fundamental role in cellular functions, including the regulation of cell signaling, gene expression, and many repair processes. The proteasome is composed of a 20S catalytic core that can function independently, or can associate with regulatory complexes, such as PA700 and PA28 [1]. The catalytic core contains four stacked rings of seven subunits each. The inner two rings are composed of the β subunits, which contain three pairs of subunits that possess proteolytic activity. In the standard proteasome, the catalytic subunits are β1, β2 and β5. A different class of β subunits [Lmp2 (β1i), MECL-1 (β2i) and Lmp7 (β5i)] can replace the catalytic β subunits of the standard proteasome to form the core of the immunoproteasome. Hybrid proteasomes, containing a mixture of standard and immunoproteasome subunits, have also been described [2], [3]. While the proteasome composition in most cells is heterogeneous, the relative ratio of different subtypes is cell-specific and can be modulated under different cellular conditions [3], [4].

The expression of immunoproteasome is well-documented in bone marrow-derived cells, where one of its functions is to generate antigenic peptides for MHC Class I occupancy for recognition by CD8 T lymphocytes [5], [6]. However, immunoproteasome expression is not limited to the immune system and has been reported in cells with a limited capacity for antigen-presentation, such as muscle, neurons, and epithelial cells [7], [8]. Furthermore, immunoproteasome is implicated in diseases associated with cardiac and skeletal muscle [7], [9], [10], retina [11], and brain [12]–[14]. Up-regulated expression of immunoproteasome is also observed in acutely injured retina and brain, and with oxidative- and cytokine-induced stress in cultured human and murine cells [8], [15]. These results suggest that immunoproteasome plays a role in responding to stress and injury [16].

Evidence from immunoproteasome knock-out (KO) mice and human diseases associated with mutations in immunoproteasome subunits corroborate the role of immunoproteasomes in the stress response, and extend its role to maintaining cellular homeostasis. Immunoproteasome KO mice exhibit decreased retinal function [17], an increased incidence of autoinflammatory diseases, and diabetes [18]–[23], and were more susceptible to the accumulation of oxidized protein products in the inflammation-challenged brain of experimental autoimmune encephalomyelitis [24]. In humans, missense and nonsense (truncation of protein) mutations in the Lmp7 subunit have been associated with various diseases characterized by autoinflammation, muscle dystrophy, and/or lipodystrophy phenotypes [18]–[21]. Additionally, patients with single nucleotide polymorphisms (SNPs) in immunoproteasome subunits (Lmp7 and Lmp2) bear higher risks for diabetes and ankylosing spondylitis [22], [23]. Taken together, these data reveal ever-expanding roles for immunoproteasome in regulating cell processes that are essential for maintaining cellular homeostasis and in responding to stress and injury.

The current study investigates how the absence of immunoproteasome affects the corneal epithelium during wound healing using immunoproteasome knockout mice that are deficient in two catalytic subunits (Lmp7/MECL-1). Corneal re-epithelialization following mechanical debridement is a widely used corneal wound healing model, with a well-defined healing processes, including the infiltration of immune cells, apoptosis and migration of keratocytes, and the proliferation and migration of corneal epithelial cells [25], [26]. p38MAPK and NFκB signaling have been shown to regulate re-epithelialization of the corneal epithelial cells [27], [28]. Our study reveals that immunoproteasome deficiency resulted in elevated apoptosis in uninjured corneas, and led to slower re-epithelialization and re-establishment of the epithelial barrier after mechanical debridement in the KO animals.

## Results

### Expression of immunoproteasome in the murine cornea

**Figure 1:**
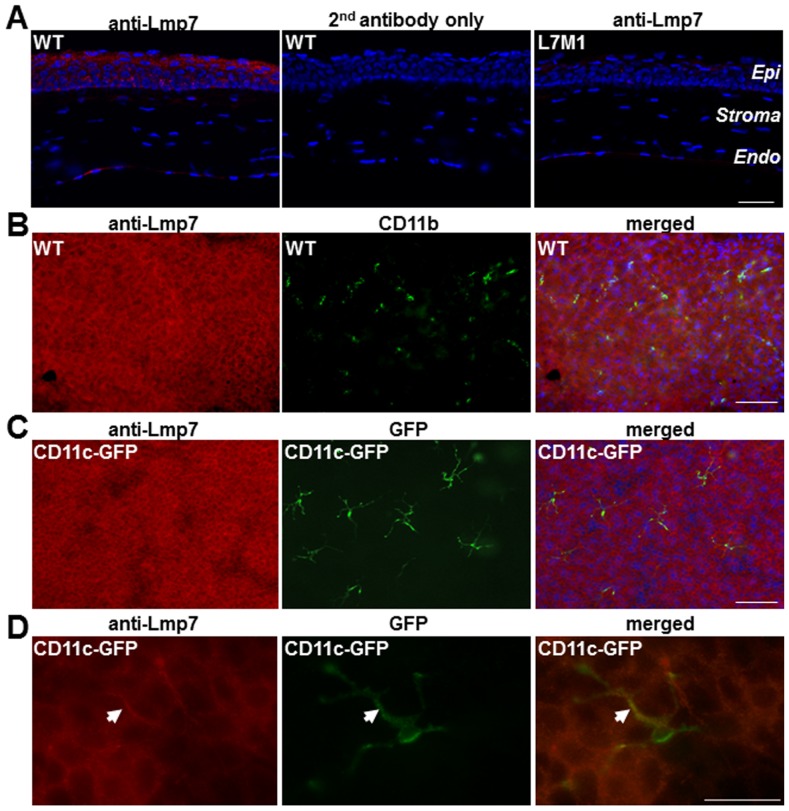
Expression of immunoproteasome in murine corneal epithelium. *(* ***A*** *)* Immunostaining with anti-Lmp7 antibody in WT corneal sections showed expression of immunoproteasome. Negative controls included staining with secondary antibody only (WT) and staining with anti-Lmp7 in immunoproteasome knock-out mice (L7M1). Red: Lmp7; Blue: DAPI-stained nuclei (100× magnification; scale bar, 50 µ). “*Epi*”: epithelium; “*Endo*”: endothelium. *(* ***B*** *)* Immune reactions of whole-mount corneas from WT mice using anti-Lmp7 (red) and anti-CD11b (green) antibodies. Images were taken at different focal depths of the same visual field in the peripheral cornea. Merged image shows anti-Lmp7 staining was not limited to the myeloid cells. Blue: DAPI-stained nuclei (100× magnification; scale bar: 150 µ). *(* ***C*** *)* Whole-mount corneas from CD11c-DTR-GFP transgenic mice stained with anti-Lmp7 antibody (red). GFP (green) fluorescence was associated with dendritic cells. Merged image showed minimal contribution of dendritic cells to the anti-Lmp7 staining. Blue: DAPI-stained nuclei (100× magnification; scale bar, 150 µ). *(* ***D*** *)* Higher magnification images of CD11c-GFP positive dendritic cells. Arrow heads indicate co-localization of Lmp7 and CD11c signals (merged). Images were taken at 400× magnification; scale bar, 50 µ.

Immunostaining of corneal sections from WT mice using an antibody against the immunoproteasome subunit Lmp7 revealed prominent staining in the epithelial layer (Figure 1A, left panel). As expected, sections from WT mice stained with the secondary antibody only (Figure 1A, middle panel) and anti-Lmp7 staining of corneal sections from L7M1 mice (Figure 1A, right panel) showed minimal background signal. These results suggest immunoproteasome was abundantly expressed in the uninjured cornea.

Bone marrow-derived immune cells, which express high levels of immunoproteasome, are also present in the cornea and may contribute to the observed Lmp7 staining signals. Previous studies have found that resident immune cells were more concentrated in the peripheral cornea and limbus, and to a much lesser extent in the central cornea [29]. In addition, immune cells were usually found in the stroma and basal layer of the epithelium but not in the superficial layer of the epithelium. In contrast to the distribution of immune cells, Lmp7 staining for immunoproteasome displayed a limbus-to-limbus, continuous distribution in the murine corneas (Figure 1A). Additionally, the Lmp7 staining was significant in the suprabasal and superficial layers of the corneal epithelium. To directly test the contribution of immune cells to the observed Lmp7 immunostaining in the corneal epithelium, whole mounts of WT cornea were double stained for Lmp7 and immune cell markers to investigate the expression and distribution of immune cells relative to the expression and distribution of Lmp7. Expression of CD11b, a cell surface marker for myeloid cells, and CD11c, a dendritic cell marker, were used to identify resident immune cells in the cornea.

Lmp7 staining was strongest in the suprabasal and superficial corneal epithelial layers (left panel, Figure 1B), while CD11b-positive myeloid cells were found in the basal layer of the corneal epithelium near the peripheral cornea (middle panel, Figure 1B). The merged image from the left and middle panels of Figure 1B (taken at a different focal depth of the same viewing field) showed that immunoproteasome expression was not limited to myeloid cells (right panel, Figure 1B). The distribution of CD11c-positive dendritic cells in the cornea was investigated using CD11c-DTR/GFP mice, in which expression of the diphtheria toxin receptor-GFP fusion protein is driven by the CD11c promoter, allowing corneal dendritic cells to be identified by their GFP fluorescence. As observed in WT mice, these transgenic mice showed prominent corneal staining with the anti-Lmp7 antibody (Fig. 1C and 1D, left panels) that included the resident dendritic cells (Fig. 1C and 1D, middle panels) as well as the surrounding epithelial cells. Co-localization of antibody and GFP fluorescent signals was most obvious in the merged image (Fig. 1D, right panel). Of note, the dendritic cell located near the basal layer of the corneal epithelium (Figure 1D), exhibited Lmp7 expression restricted to the dendrites that inserted between the epithelial cells. Together, these co-localization experiments clearly showed that while corneal immune cells expressed immunoproteasome, they made a minor contribution to corneal staining for immunoproteasome.

**Figure 2:**
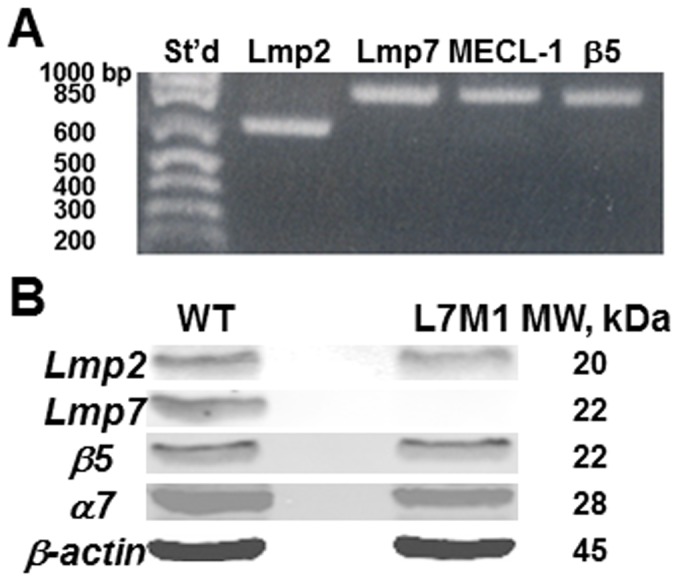
Expression of immunoproteasome and standard proteasome in WT and L7M1 mice. *(* ***A*** *)* RT-PCR of mRNA extracted from the corneal epithelium of WT mice. *(* ***B*** *)* Western blot of corneal epithelial lysates probed with anti-Lmp2, anti-Lmp7, anti-β5, anti-α7 and anti-β-actin antibodies.

In addition to the immunostaining results, RT-PCR and Western blots also confirmed the expression of immunoproteasome in epithelial cells scraped from WT corneas. As shown in Figure 2A and 2B, WT cornea expressed the immunoproteasome subunits Lmp7, Lmp2 and MECL1 (RT-PCR results in Figure 2A), and was confirmed by Western blots for the presence of Lmp7 and Lmp2 proteins (Figure 2B). As expected, the L7M1 KO mice were null for Lmp7 subunit but not Lmp2 (Figure 2B). The standard proteasome subunit β5 and the non-catalytic subunit α7, which is present in all 20S cores, were present in both WT and L7M1 corneas (Figure 2B). RT-PCR of mRNA isolated from WT corneal epithelial explant cultures also showed expression of immunoproteasome (data not shown).

### No difference in morphology in the uninjured cornea

**Figure 3:**
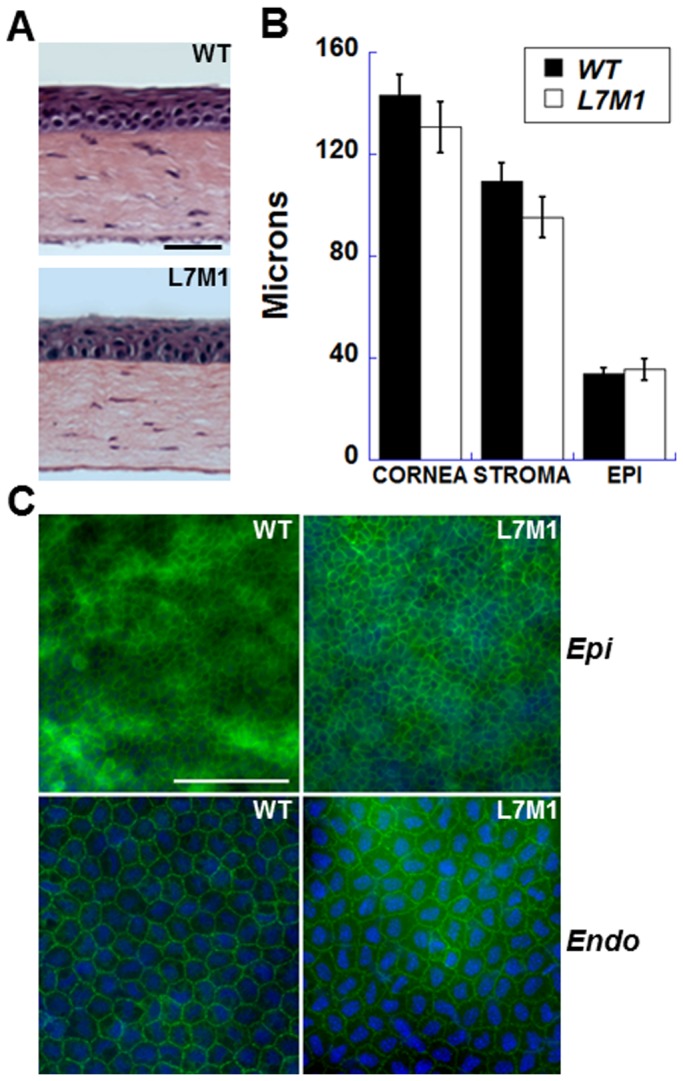
Uninjured corneas of L7M1 mice exhibited no gross anatomical differences compared to WT mice. *(* ***A*** *)* H&E stained cross sections of the uninjured WT and L7M1 mouse corneas. (Scale bar, 50 µ) *(* ***B*** *)* Comparison of corneal layer thicknesses for WT and L7M1 mice. The thicknesses of the central cornea, and the stromal and epithelial layers in the central corneal region were measured from H&E stained sections of WT and L7M1 corneas from mice of 2–3 months old. For each strain, data from five animals was expressed as mean ± SEM. *(* ***C*** *)* Representative images of WT and L7M1 corneal whole mounts stained with anti-ZO-1 antibody (100× magnification; Scale bar, 100 µ). Images of the epithelium (“*Epi*”) and endothelium (“*Endo*”) were taken at different focal depths within the same section.

Examination of bright field images of H&E stained corneal sections revealed no gross anatomical difference between WT and L7M1 mice (Figure 3A). To quantitatively assess potential differences in corneal morphology, the thickness of the cornea and corneal layers (stroma and epithelium) was measured in the central cornea of WT and L7M1 mice. As shown in Figure 3B, there was no significant difference in the thickness of the whole cornea or individual layers between WT and L7M1 mice.

To examine the integrity of the cells in the epithelial and endothelial layers, corneal whole mounts were incubated with an anti-Zona Occludens protein 1 (ZO-1) antibody and 4′,6-diamidino-2-phenylindole (DAPI) to stain nuclei. There was no difference in overall appearance of the cells from WT and L7M1 mice. Cells in the epithelial (basal layer was shown) and endothelial layers exhibited well-defined cell borders, suggesting the presence of tight junctions (Figure 3C). These results show that in the absence of injury, corneal morphology in WT and L7M1 mice was not different.

### Elevated apoptosis in the epithelium of L7M1 cornea

**Figure 4:**
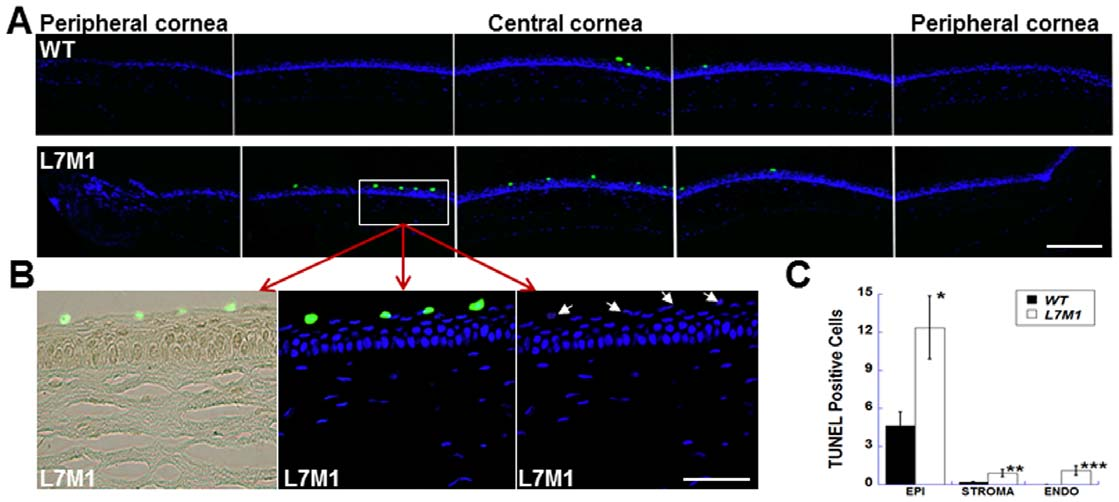
TUNEL staining revealed more apoptosis in uninjured L7M1 corneas. *(* ***A*** *)* Images of corneal sections stained by TUNEL indicated more apoptotic cells in the epithelium of L7M1 mice than of WT mice (Green: TUNEL-positive cells; 25× magnification; scale bar, 150 µ). *(* ***B*** *)* The enlarged TUNEL/bright field (left panel), TUNEL/DAPI (middle panel) and DAPI (right panel) images of the boxed region from L7M1 cornea in (A). The corresponding nuclei of TUNEL-positive cells are indicated by the arrows. (Scale bar, 50 µ) *(* ***C*** *)* Summary of TUNEL-positive cells for each corneal layer. Data are mean ± SEM (n = 10 WT; n = 12 L7M1). *p = 0.014; **p = 0.043; ***p = 0.017.

We previously observed a significant increase in apoptotic cells in the retina of L7M1 mice despite the seemingly normal gross retinal anatomy [15]. To determine if an analogous situation occurs in the cornea, sections were stained with TUNEL and the number of nuclei undergoing apoptosis across the entire arc of the cornea were counted for each corneal layer. The results show significant apoptosis in the corneal epithelia of L7M1 mice compared to WT mice (Figure 4A). L7M1 corneas have three times as many TUNEL-positive cells present in the epithelium as do WT corneas (Figure 4C). Most of these apoptotic cells were located in the superficial corneal epithelial layer (Figure 4B, the enlarged images of the boxed area in Figure 4A) where Lmp7 staining was also strong (Figure 1A). Although apoptosis was rare in both the stromal and endothelial layers, L7M1 mice had significantly higher numbers of apoptotic cells than WT mice in these layers (Figure 4C).

### Significant cell death and higher caspase-3 activity of L7M1 corneal epithelial cells *in vitro*

**Figure 5:**
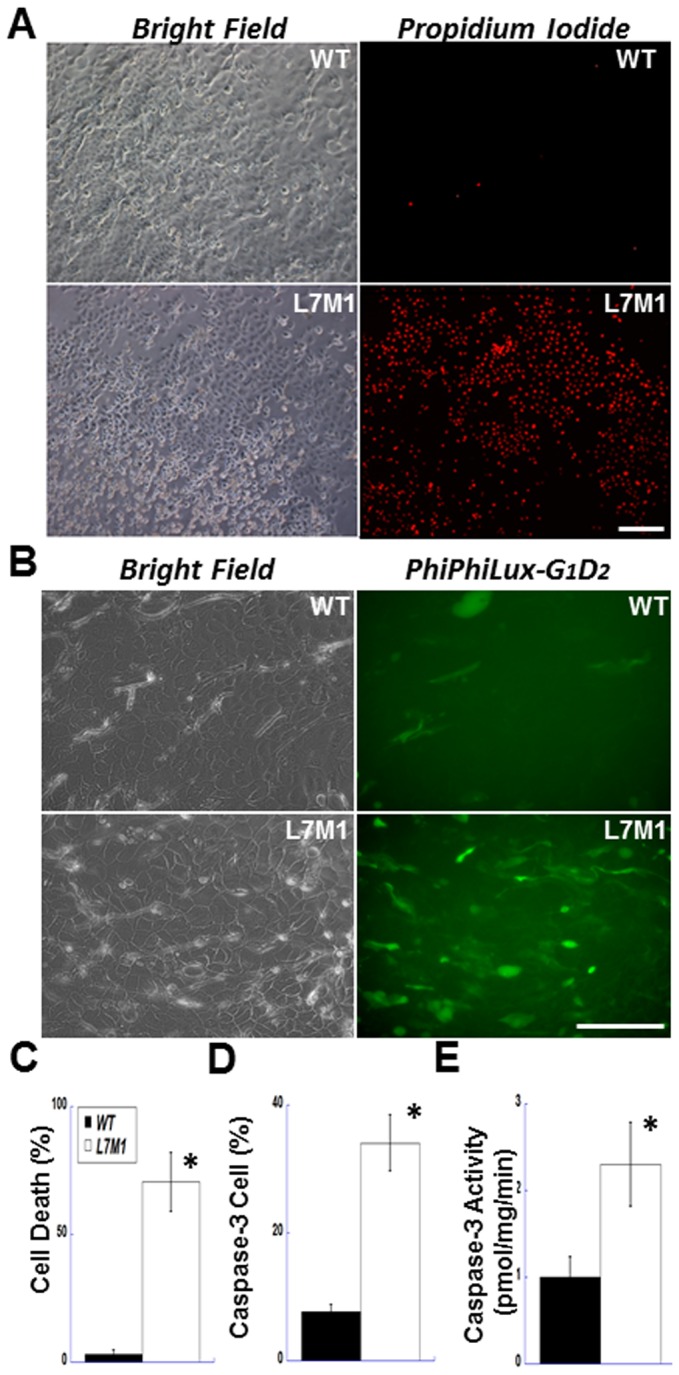
More cell death and higher caspase-3 activity observed in explant-cultured corneal epithelial cells from L7M1 mice. *(* ***A*** *)* Explant-cultured corneal epithelial cells were stained with propidium iodide on day 7 (right panels, 100× magnification; scale bar, 150 µ). The bright field images of the same viewing field are shown in the left panels. *(* ***B*** *)* After 5–6 days of explant culture, WT and L7M1 corneal epithelial cells were stained with a caspase-3 fluorogenic substrate, PhiPhiLux-G_1_D_2_ (Green; FITC; 200× magnification; scale bar, 100 µ). *(* ***C*** *)* Summary of the percentage of the PI-positive stained cells in WT and L7M1 cultures. Data are mean ± SEM (n = 11), *p<0.0001. *(* ***D*** *)* Summary of the percentage of cells stained positive for cleaved PhiPhiLux-G_1_D_2_. n = 5 WT; n = 9 L7M1; *p = 0.009. *(* ***E*** *)* Summary of caspase-3 activity determined from the cleavage of AC-DMQD-AMC in cell lysates of WT and L7M1 corneal epithelial cells. n = 6 WT; n = 4 L7M1; *p = 0.027.

The higher frequency of apoptosis in the corneal epithelium of L7M1 KO mice prompted further investigation of the epithelial cells using explant cultures. The first several days of epithelial cell outgrowth from corneal explant cultures were similar between corneas from WT and L7M1 mice. Outgrowth was initially active and composed almost exclusively of cells exhibiting cuboidal epithelial morphology. By day 6, cell detachment was noted in the L7M1 but not WT corneal explant cultures. By day 7, the epithelial cells in the L7M1 corneal explant cultures (Figure 5A, “Bright Field” lower left panel) were distinguished from WT controls (Figure 5A, “Bright Field” upper left panel) by their detachment and cell death. The difference in cell death between WT and cultures deficient in immunoproteasome was confirmed by propidium iodide staining (Figure 5A, “Propidium iodide” right panels). Approximately 70% of the cells in L7M1 corneal explant cultures stained positive with PI, compared to only 3% of cells in WT cultures (Figure 5C). After two weeks of *in vitro* culture, no living epithelial cells were found in L7M1 corneal cultures, whereas most epithelial cells from WT corneas remained viable.

To determine if cell death in L7M1 explant cultures was an apoptotic event, we measured caspase-3 activity using two different assays. Figure 5B (right panels) shows the fluorescence images of WT and L7M1 corneal explants treated with a cell-penetrating fluorogenic substrate of caspase-3, PhiPhiLux-G_1_D_2_. A significantly higher percentage of cells from L7M1 cultures than from WT cultures (34% versus 7%) were caspase-3 positive (Figure 5D). We also examined caspase-3 activity in cell lysates collected from corneal explant cultures at days 5 to 6 using the fluorogenic caspase-3 substrate AC-DMQD-AMC. Our results indicated that caspase-3 activity was more than two-fold higher in L7M1 lysates than in WT lysates (Figure 5E). These results suggest a role for immunoproteasome in protecting cells from apoptotic cell death.

### Corneal wound healing was affected in L7M1 KO mice

**Figure 6:**
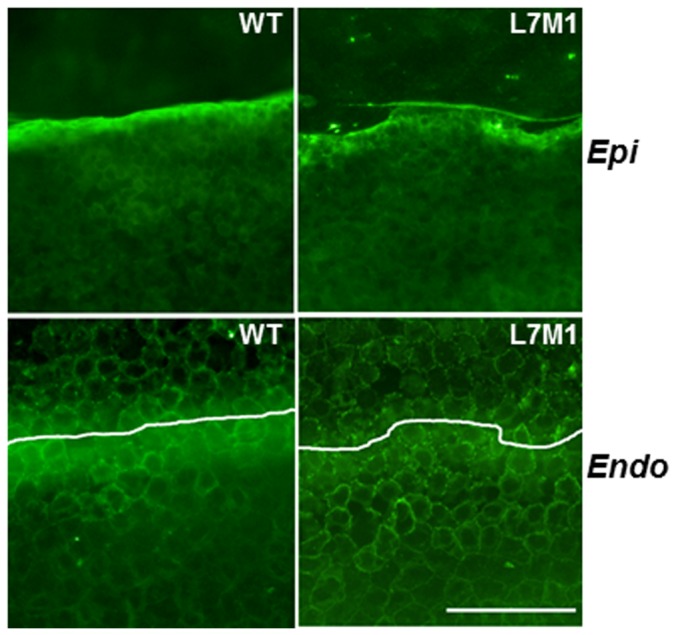
ZO-1 staining of cornea immediately after debridement (0 hour). Top panels showed the edge of debridement in the epithelium (“*Epi*”), and the endothelial layer (“*Endo*”) immediately below the area of debridement. White lines on the *Endo* figures show the boundary of the debridement in the epithelial layer. (Scale bar, 50 µ).

In addition to its role in protecting from apoptosis, immunoproteasome may function in the response to stress and injury, as we showed in the retina and brain [8], [15]. To investigate how the absence of immunoproteasome affected the injury response in cornea, we conducted a wound healing experiment by mechanical debridement of the corneal epithelium and compared the re-epithelialization process. As shown by ZO-1 staining (Figure 6), this method selectively removes the epithelial layer but leaves the endothelial layer intact.

**Figure 7:**
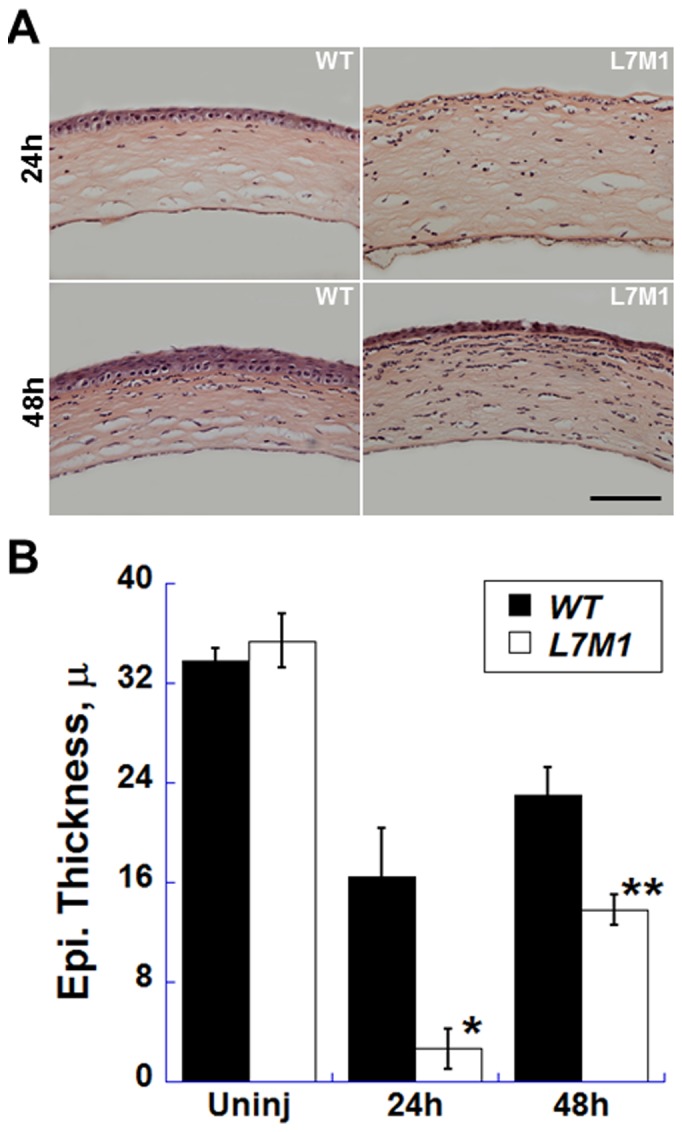
Compromised re-epithelialization of the corneal epithelium in L7M1 mice. *(* ***A*** *)* H&E- stained cross sections of the WT and L7M1 mouse corneas 24 and 48 hours after mechanical debridement. (Scale bar, 100 µ) *(* ***B*** *)* The central corneal epithelial thickness was measured in uninjured corneas and at 24 and 48 hours after debridement. Data are mean ± SEM. (n = 4 WT; n = 5 L7M1), *p = 0.002; **p = 0.006.

**Figure 8:**
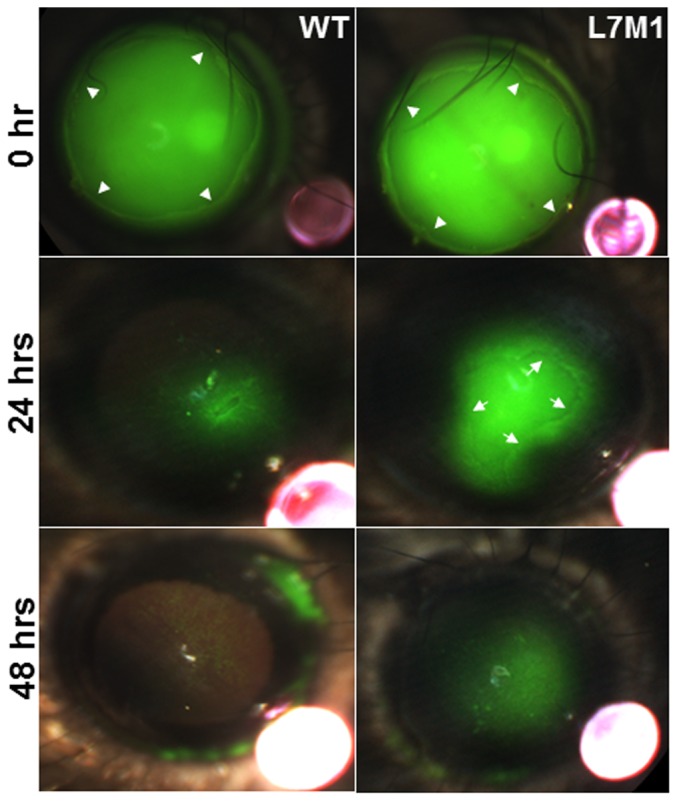
The epithelial barrier function was affected in the debrided L7M1 corneas. Fluorescein-stained mouse corneas imaged at 0, 24 and 48 hours after mechanical debridement. The boundary of the mechanically debrided epithelium was indicated by the arrowheads (“*0 hr*”, upper panels). The central regions in L7M1 cornea were not completely closed at 24 hrs, and the boundary of the regrown epithelium is indicated by the arrows near the pericentral region (“*24 hrs*”, middle panels). The red objects in the lower right corner in each photograph were ruby spheres (0.5 mm diameter). When illuminated in lower light intensity, these spheres were used as the size reference for image analysis to determine the size of the injury.

The debrided corneas (Figures 7 and 8) showed partial corneal healing at 24 hours. Examination of H&E stained corneal cross sections revealed that at 24 hours, the epithelium of the WT corneas had already re-populated most of the debrided area in the central cornea and displayed stratified cell layers (Figure 7A, upper left panel). In contrast, the debrided area in the central cornea of L7M1 mice remained exposed (Figure 7A, upper right panel). Also note that the slower re-epithelialization in L7M1 corneas permitted corneal swelling that persisted at 24 hours after debridement due to the loss of the epithelial barrier (Figure 7A). Although the debrided corneal surface was covered by epithelial cells at 48 hours for both WT and L7M1, the proliferation and stratification of the corneal epithelia in L7M1 mice was significantly less than the WT, as shown by the significantly thicker epithelial layer in WT at both 24 and 48 hours (Figure 7B, p<0.05). At 48 hrs, WT cells had recovered to 75% of the corneal thickness, whereas L7M1 were only 50% recovered with noticeable corneal swelling.

In addition to the proliferation and stratification of the corneal epithelia, the epithelial barrier function during the wound healing was also evaluated by the staining with fluorescein, which is excluded from areas where the barrier is intact and conversely, penetrates areas where there is a loss in epithelial barrier integrity. The stained area in WT corneas (25±9%, mean ± S.E.M.) at 24 hours was reduced compared to L7M1 KO mice (49±14%) (Figure 8), which showed a larger defect in epithelial barrier function. In addition, the boundary of epithelial regrowth can still been seen (indicated by the arrows in Figure 8, middle right panel). By 48 hours, fluorescein staining was reduced significantly in WT corneas, suggesting recovery of epithelial barrier function. However, fluorescein staining was still observed in KO corneas, suggesting an incomplete barrier remained at 48 hrs.

**Figure 9:**
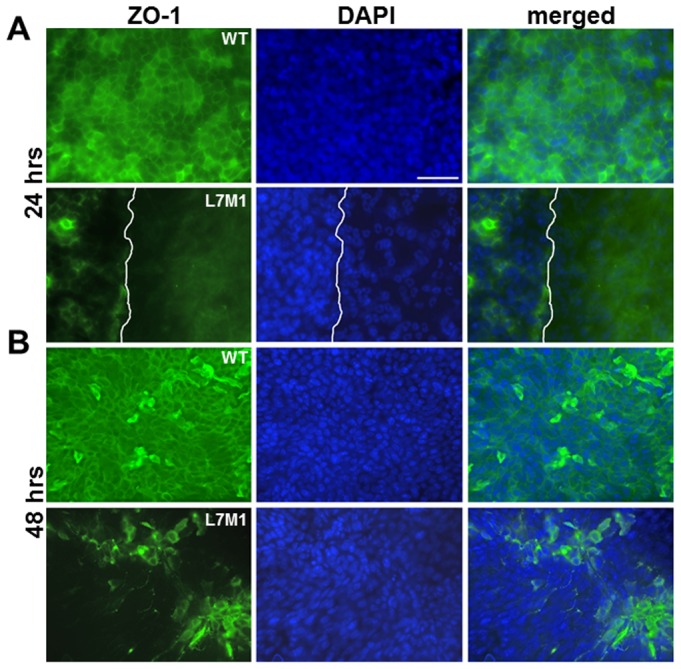
The expression of ZO-1 was affected in the debrided L7M1 corneal epithelia. ZO-1 (green) and nuclei (DAPI, blue) staining of the central cornea at 24 hours *(* ***A*** *)*, and 48 hours *(* ***B*** *)* after debridement. (Scale bar, 50 µ) The images at 24 hours were taken near the pericentral cornea where the epithelium has regrown in WT but not in L7M1. The white line in L7M1 indicates the boundary of the regrown epithelium (left) and area devoid of epithelial cells (right).

The epithelial barrier is maintained by tight junctions that form between cells and involve a number of proteins, including ZO-1. To confirm potential defects in formation of tight junctions in L7M1 corneas, whole mounts were stained with anti-ZO-1 antibody at 24 and 48 hours. At 24 hours, the central cornea of L7M1 KO mice had not re-epithelialized and thus, no or at best, limited epithelial cells were present. Therefore, we examined the pericentral corneal regions where the boundary of regrown epithelia (indicated by the white line, “L7M1”, Figure 9A) can be clearly identified. Epithelial cells at the margins of the re-epithelialization (left side of the white line, “L7M1”, Figure 9A) contained spotty ZO-1 staining, even though DAPI-stained nuclei showed that cells had repopulated this region. In comparison, the corresponding pericentral corneal region for WT at 24 hours showed a uniform distribution of ZO-1 positive cells with well-defined cell borders (“WT”, Figure 9A). In the central cornea at 48 hrs, DAPI and ZO-1 staining demonstrated that most of the cell borders contained tight-junctions in WT corneas (“WT”, Figure 9B). For L7M1 corneas, DAPI staining revealed a uniform distribution of epithelial cells. However, these cells largely failed to express ZO-1 (“L7M1”, Figure 9B). These results confirm major differences in corneal wound healing in WT and immunoproteasome-deficient mice. Cells in WT epithelia had defined ZO-1 staining in the pericentral cornea at 24 hours, and both pericentral and central cornea at 48 hrs post injury, with recovery of the epithelial barrier function by 48 hrs. In contrast, corneas deficient in immunoproteasome demonstrated defects in formation of tight junctions, which could explain the observed incomplete epithelial barrier function that remained at 48 hrs post-injury.

### Production of the inflammatory cytokines in the debrided corneas of L7M1 KO mice

**Figure 10:**
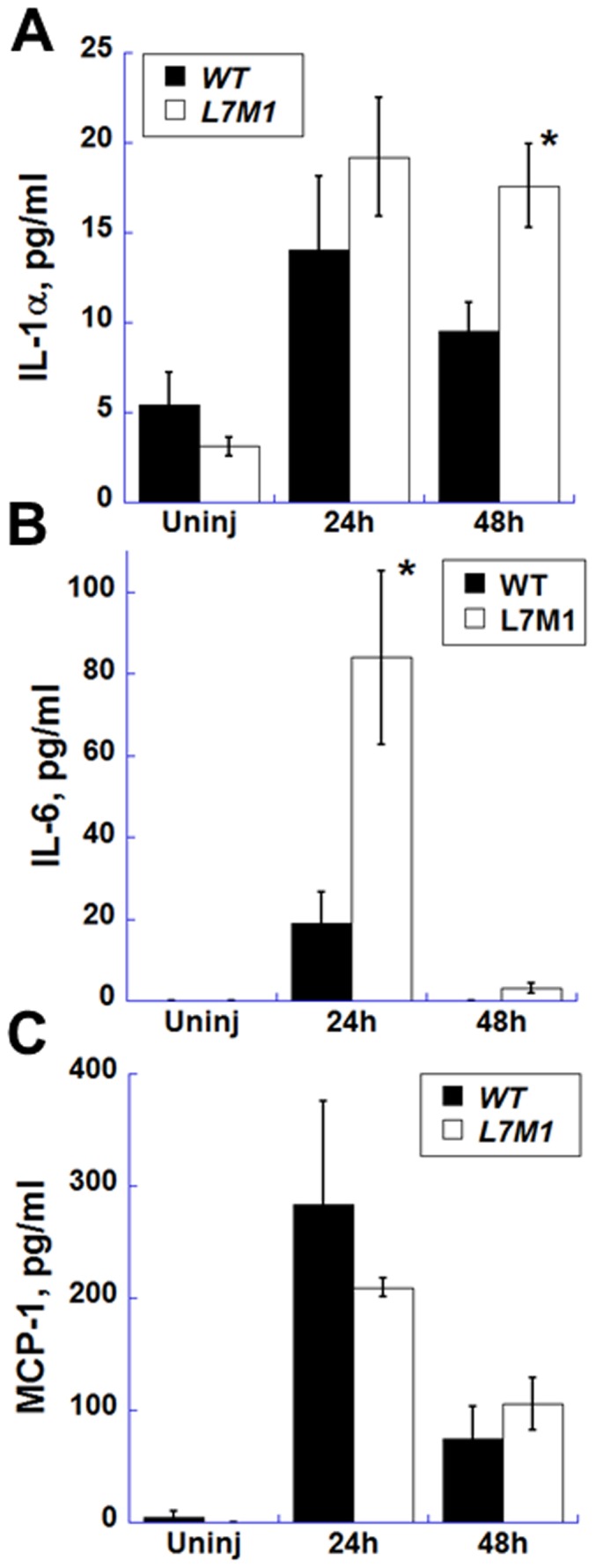
Production of inflammatory cytokines in the debrided WT and L7M1 corneas. WT and L7M1 mice were analyzed for the cytokines IL-1α *(* ***A*** *)*, IL-6 *(* ***B*** *)* and MCP-1 *(* ***C*** *)* production in uninjured (“*Uninj*”) cornea and at 24 and 48 hrs after debridement using Cytometric bead arrays. (n = 3 for each group).

To begin defining a mechanistic explanation for the delayed re-epithelialization and re-establishment of barrier function in the debrided cornea in L7M1 mice, the expression of several signaling molecules and cytokines involved in corneal wound healing was measured. Cytometric bead assays for IL-1α, IL-1β, IL-6, and monocyte chemotactic protein-1 (MCP-1) were used to measure the total content of cytokines, which includes both secreted and intracellular proteins, in the lysates from dissected cornea. While IL-1α was in low abundance in the uninjured murine cornea, its expression level was higher in the L7M1 KO mice both at 24 hours and 48 hours after debridement (Figure 10A, p = 0.04 at 48 hr). IL-6 was undetectable in uninjured corneas, but a significant increase was observed 24 hours after corneal debridement; IL-6 levels were four-fold higher in L7M1 corneas compared to WT (Figure 10B, p = 0.04). At 48 hours, the level of IL-6 returned to baseline (Figure 10B). The expression of MCP-1 increased significantly with injury, but the levels were not different between WT and L7M1 corneas (Figure 10C). The expression of IL-1β was not detectable in the murine cornea in our assays (data not shown).

## Discussion

The cornea encounters constant environmental insults, including ultra-violet light, osmolarity fluctuation, pathogens and injuries. Since vision is dependent on corneal avascularity and transparency, efficient and active coping mechanisms are required to maintain corneal integrity and function. The expression of immunoproteasome in normal cornea appears to contribute to the cytoprotective mechanisms in the cornea, as immunoproteasome deficiency compromised the survival stress response and led to greater cell death and slower wound healing.

We observed significantly more apoptosis of corneal epithelial cells from immunoproteasome KO mice both *in vivo* (more frequent apoptotic cells in the stroma and endothelium) and *in vitro* (greater caspase-3 activity). Our results are consistent with a recent study with Lmp7-deficient cells where enhanced caspase-3/7 activity was observed under cytokine-induced oxidative stress, or when treated with the apoptosis inducer etoposide [24]. While it is possible that the immunproteasome deficiency of resident bone marrow-derived cells may contribute to the observed corneal phenotype *in vivo*, CD11b-positive cells were not found in the outgrowths of our corneal explant cultures by CD11b immunostaining (data not shown). Accordingly we propose that the increased apoptosis of L7M1 corneal epithelial cells was a direct consequence of immunoproteasome deficiency.

Data from cells and tissues deficient in immunoproteasome also provide compelling evidence for a critical role in protecting from oxidative stress. A recent report from our lab showed cultured retinal pigment epithelial cells from L7M1 KO mice were more susceptible to peroxide-induced cell death compared with WT cells [15]. In Lmp7-deficient cells, an increase in apoptosis was attributed to the compromised ability of immunoproteasome-deficient cells to process defective ribosomal products (DRiPs) that contain an abundance of oxidized and misfolded proteins [24]. Tissues from Lmp2 knock-out mice showed an accumulation of oxidized proteins, suggesting that immunoproteasome's critical role in surviving an oxidative insult includes more efficient degradation of oxidized proteins [30]. Since greater oxidative stress has been demonstrated in the superficial epithelium compared with basal layers of the cornea [31], the data from KO mice demonstrating a role for immunoproteasome in coping with oxidative damage suggest a possible explanation for the localized high immunoproteasome expression in the corneal epithelium.

In addition to its capacity to degrade oxidized protein substrates, immunoproteasome may also regulate stress-related pathways, such as NFκB signaling. NFκB signaling is activated by oxidants and ligands to toll-like and TNF receptors, and has been demonstrated to regulate both pro- and anti-apoptotic events [32]. In the corneal epithelium, ultraviolet (UV) radiation is a major environmental insult that can up-regulate NFκB signaling, which is necessary for the stress-related response [33]. NFκB signaling can be activated via (1) limited proteolysis of the precursors of NFκB family members (such as p100/p105), or (2) degradation of an inhibitory sequestering protein, IκB, which binds and prevents NFκB from entering the nucleus. Both types of proteolytic processing are accomplished by the proteasome. Studies on cultured lymphocytes from Lmp2 KO mice revealed defects in proteolytic processing of both NFκB precursors and the inhibitory regulator IκB, suggesting a direct contribution for the immunoproteasome in Lmp2 KO mice [34]. An important regulatory mechanism built into the NFκB pathway is the induction of multiple inhibitors (i.e., IκBα, A20) that serve to attenuate or shut down signaling. Therefore, a potential consequence of altered signaling is the decreased production of negative feedback molecules that preventing chronic NFκB signaling and consequent overproduction of proteins, such as IL-6. Cells with immunoproteasome deficiency may therefore become more vulnerable to stress due to dysregulated NFκB signaling and the subsequent altered gene expression required for survival.

Dysregulated NFκB signaling in the immunoproteasome deficient cornea may also affect corneal wound healing via other signaling pathways, such as p38MAPK signaling, which is also essential for the corneal re-epithelialization process [27]. The cross-talk of p38MAPK and NFκB is well-documented in various tissues and cells [35]–[37] and occurs through the direct interaction of IκB kinase β(IKKβ), with p38MAPK [28]. The p38MAPK pathway can also be activated by the accumulation of oxidized proteins. Importantly, the direct consequence of NFκB and p38MAPK activation is the upregulation of multiple cytokines, such as IL-6. Results from the current study show increased IL-6 cytokine production in immunoproteasome-deficient corneas, suggesting aberrant regulation of one or more pathways involved in the stress response. These results are consistent with reports from human patients with Nakajo-Nishimura syndrome (LMP7 mutation) that have abnormally high level of IL-6 in serum, cells (skin or B cells) and cultured fibroblasts [20], [21], [38]. It was suggested that the observed hyperproduction of IL-6 was caused by the accumulation of ubiquitinated and oxidated proteins due to immunoproteasome dysfunction [38].

In addition to increased IL-6, we also observed a significantly higher level of IL-1α in the debrided L7M1 corneas. Both IL-1α and IL-6 were shown by immunostaining to be localized to the regenerating basal epithelial cells during injury [39]. IL-1α is secreted by corneal epithelial cells as a key factor for stromal fibroblast activation, and also by stromal fibroblasts in an autocrine manner to stimulate their proliferation and differentiation. As an inflammatory cytokine, IL-6 is secreted by corneal epithelial cells and fibroblasts to promote the infiltration of inflammatory cells. Others have shown that IL-6 promoted epithelial wound healing of debrided corneas in rabbits [40], was essential for the corneal sterile inflammation and wound healing in mice [41], and facilitated the migration but not the proliferation of cultured epithelial cells [42], likely via a fibronectin-dependent mechanism [39]. Therefore, IL-6 is required for optimal wound healing. However, the compromised corneal epithelial recovery in L7M1, despite the higher cytokines levels, suggests potential disruption in other processes downstream of IL-6 signaling or those involved in epithelial secretion of IL-6. Since our measurement of total content of IL-6 included both intracellular and secreted protein, future experiments are needed to distinguish the mechanism behind the discrepant results of higher IL-6 and delayed wound healing.

The high IL-6 production in the injured L7M1 cornea may negatively impact the re-establishment of epithelial barrier function. A recent study showed that high levels of IL-6 significantly reduced the expression of ZO-1, altered its localization pattern and disrupted the epithelial barrier function in a human epithelial cell line, HCE-2 [43]. These results may help explain the observed decreased ZO-1 staining and formation of the tight junction observed in the debrided L7M1 corneas.

In summary, we have shown substantial immunoproteasome expression in corneal epithelial cells. Immunoproteasome-deficient epithelial cells displayed more apoptosis in corneal explant cultures and in intact corneas, suggesting a potential contribution of the immunoproteasome in corneal homeostasis. We have also, for the first time, demonstrated that deficiency in immunoproteasome may also lead to delayed epithelial wound healing in the mechanical debridement mouse model. In light of these results and the recent discoveries that immunoproteasome provides cytoprotective effects in the retina and brain under stress and injury, a comprehensive study to investigate the novel roles of immunoproteasome in the cornea bears significant merit.

## Materials and Methods

### Mice

Corneal tissues from C57BL/6 (wild-type, WT) and double knock-out (*lmp7^−/−^/mecl-1^−/−^*, L7M1) mice from 2 to 3 months of age were used for experiments. Breeders for the immunoproteasome-deficient mice on the C57BL/6 genetic background were provided by J.J. Monaco (University of Cincinnati). L7M1 mice show no gross physical abnormalities and breed well. Descriptions of gene deletions, mouse characteristics and retinal functions have been previously published [17], [44]. CD11c-DTR/GFP transgenic mice carry a transgene with diphtheria toxin receptor cDNA fused with GFP cDNA driven by the CD11c promoter as described [45]. Mice produced in our colonies at the University of Minnesota were housed in an animal facility maintained at 20°C with a 12-hour light and dark cycle, and handled according to the guidelines of the Institutional Animal Care and Use Committee of the University of Minnesota and the National Institutes of Health. Animal procedures conformed to the ARVO Statement for the Use of Animals in Ophthalmic and Vision Research.

### Immunofluorescent staining of corneal sections and whole corneas

To prepare immunostainings of corneal sections, eyes from WT and L7M1 mice (2 to 3 months old) were enucleated and immediately snap-frozen in Tragacanth (Sigma-Aldrich Co., Saint Louis, MO, USA). Sections were cut 12 µm thick on a LEICA CM 3000 cryostat, mounted and air dried on Fisher Colorfrost Plus Microscope slides (Fisher Scientific, Pittsburg, PA, USA). Before antibody labeling, tissue sections were fixed in ice-cold acetone for 15 minutes and washed in PBS. Endogenous peroxidase was inactivated by application of 0.3% hydrogen peroxide. The sections were incubated with 10% donkey normal serum for 30 minutes to block non-specific immunoglobulin binding. The corneal sections were further incubated overnight with a polyclonal rabbit anti-LMP7 (1∶500, Abcam, Cambridge, MA, USA) antibody. After three rinses with PBS, Rhodamine- (1∶200, Jackson ImmunoResearch, West Grove, PA, USA) labeled secondary antibody was applied to sections and incubated for 3 hours. The slides were cover slipped with mounting medium containing DAPI (Vector, Burlingame, CA, USA), and the bright field and fluorescence images were taken with a LEICA DM 4000B using Leica Application Suite version 3.8 (Leica Microsystem, Wetzler, Germany).

For immunostaining of the whole mount mouse corneas, mice were perfused with PBS plus heparin (1 unit/mL) following euthanasia. The eyes were enucleated and fixed in 4% paraformaldehyde. The corneas were excised and incubated in 20 mM EDTA for 30 minutes at 37°C, and blocked in PBS/0.2% TritonX-100/1% BSA for one hour at room temperature. Anti-Lmp7, anti-CD11b (1∶400; BD Biosciences), anti-GFP (1∶400; BD Biosciences) or anti-ZO-1 (1∶400; Sigma) antibodies were prepared in the blocking solution and incubated with the mouse corneas overnight at 4°C. The corneas were then washed with PBS, incubated with the appropriate secondary antibody for 3 hour at room temperature, and counterstained with DAPI. The whole mount corneas were cover slipped and visualized as described above.

### Western blot experiments

Mouse corneal epithelial cells were scraped from dissected corneas with a scalpel. The collected epithelial cells were lysed in RIPA buffer, and the protein concentrations of these lysates were determined using a BCA Protein Assay kit (Pierce, Rockford, IL, USA). Twenty µg total protein from each sample was separated on 16% SDS-PAGE gels, transferred onto nitrocellulose membranes, and probed with rabbit anti-Lmp2, rabbit anti-Lmp7, mouse anti-α7 (Biomol International, Plymouth Meeting, PA, USA) and rabbit anti-β5 (Abcam, Cambridge, MA, USA) polyclonal antibodies. Membranes were also probed with a mouse anti-β-actin monoclonal antibody (Sigma) to ensure equal loading. Primary antibodies were detected by secondary antibodies labeled with IR dyes, IRDye™800 anti-Rabbit IgG and IRDye™700 anti-mouse IgG (Rockland, Gilbertsville, PA, USA), respectively. Protein bands were imaged and digitized with an Odyssey Infrared Imager and accompanying software (LI-COR, Lincoln, NE, USA).

### RT-PCR

Total RNA was extracted from scraped corneal epithelium of dissected corneas with an RNeasy kit (Qiagen, Valencia, CA, USA) per the manufacturer's instructions, then reverse-transcribed to cDNA using a Superscript III First-Strand Synthesis kit (Invitrogen, Carlsbad, CA, USA), according to the manufacturer's instructions. PCR was performed and the products were separated by electrophoresis on 1.2% agarose gels containing ethidium bromide and visualized under UV light. The amplicons were validated by automatic sequencing method performed at the Microchemical Facility at the University of Minnesota. (Primers are listed in Table 1).

**Table 1:** Sequences of the oligonucleotide primers used in the RT-PCR experiments.

Primer	Sequence (5′-3′)	Size of amplicon (base pair)	Annealing temperature (°C)
mLmp2_F	AACATATGCTGCGGGCAGGAGCACC		
mLmp2_R	AAGGATCCTCACTCATCGTAGAATTTTGG	660	61
mMECL-1_F	AACATATGCTGAAGCAGGCAGTGGAAC		
mMECL-1_R	AACTCGAGTCATTCCACCTCCATGGCCTG	822	64
mLMP7_F	AACATATGGCGTTACTGGATCTGTG		
mLMP7_R	AAGGATCCTCACAGAGCGGCCTCTCCGTAC	831	60
mβ5_F	AACATATGGCGCTGGCCAGCGTGTTG		
mβ5_R	AAGGATCCTCAGGGGACAGATACACTACTG	795	64

### Measurement of the thickness in the central corneas

Whole globes were fixed in 10% phosphate-buffered formalin then paraffin-embedded and sectioned sagitally. High resolution digital images of the hematoxylin and eosin (H & E) stained sections of the central corneas (taken through the optic nerve, the central cornea was defined as the area of the cornea directly anterior to the optic cup) were captured at 200× magnification with a Leica DMR microscope. To determine the corneal thicknesses, BIOQUANT Life Science 2009 V9.7.6 software (BIOQUANT Image Analysis Corp, Nashville, TN) was used. The averaged results from two sections of each animal were used for this study; each section was measured in three separate areas within the central corneal region.

### Corneal explant culture

Corneas were dissected from whole globes and cut into 1×1 mm blocks. The corneal blocks were cultured for epithelial outgrowth using growth medium composed of DMEM/Ham's F12 medium (Mediatech Inc., Manassas, VA), 10% fetal bovine serum (FBS), 1× ITS media supplement (Sigma), 0.5 µg/mL hydrocortisone, 2 ng/mL epidermal growth factor, 0.5% dimethyl sulfoxide, 50 µg/mL penicillin/streptomycin, 1.25 µg/mL amphotericin B, and 0.1 µg/mL epinephrine. Cultures were incubated at 37°C in a humidified atmosphere containing 5% CO_2_. Culture medium was changed every 2 days.

### Cell death and apoptosis assays

To analyze cell death *in vitro*, we used propidium iodide to stain dead or dying cells in corneal explant cultures. On day 7, cultures were incubated with PI in serum-free medium (SFM) for 5 minutes followed by extensive washing in SFM. The numbers of PI-positive cells were determined using a Zeiss Axiovert 200M epifluorescence microscope with AxioVision Rel. 4.5 software. To determine the total cell numbers within the same viewing area, cells were permeabilized with 1% TX-100, washed with SFM, and then stained a second time with PI. The percentage of cell death was calculated from the ratio of dead cells to total cells.

To quantify cellular apoptosis *in vitro*, two methods were used: (1) PhiPhiLux-G_1_D_2_ (OncoImmunin, Gaithersburg, MD, USA) fluorogenic substrate for caspase-3 was added to cell cultures at day 6 and incubated for one hour. The substrate was then removed, the cells were washed twice in SFM, and the fluorescence signal from the cleaved substrate was observed using an epifluorescence inverted microscope. (2) The fluorogenic substrate AC-DMQD-AMC (100 µM, Enzo Life Sciences, Plymouth Meeting, PA) was used to measure caspase-3 activity in L7M1 and WT cell lysates. The buffer contained 0.1 M HEPES (pH 7.4), 0.2% CHAPS, 20% sucrose, 2 mM EDTA, 200 mM NaCl, and 20 mM DTT. Duplicates of individual samples were used in this assay. The measured caspase-3 activity was normalized to WT.

To investigate apoptosis of epithelial cells in vivo, corneal sections (12 µm thick) from WT and L7M1 mice were analyzed by TUNEL using a fluorescence In Situ Cell Death Detection kit (Roche Diagnostics, Indianapolis, IN, USA). TUNEL-positive cells in each corneal layer (epithelium, stroma and endothelium) were counted for the entire corneal cross section. The results were obtained by averaging the TUNEL-positive cells from four sections per eye per animal.

### Corneal epithelium debridement

Mice were anesthetized by subcutaneous injection of ketamine/xylazine. Drops of proparacain (0.5%, Alcon) were applied to the corneas as a topical anesthetic. A 2 mm area was marked on the surface of one cornea with a biopsy punch (Miltex, York, PA, USA), then debrided with a micro drill fitted with a 0.5 mm carbon steel burr (Fine Science Tools, Foster City, CA, USA) [27], [46]. The other eye was left uninjured. Antibiotic ophthalmic ointment was applied to both eyes (Neomycin and Polymyxin B Sulfates and Bacitracin Zinc, Bausch and Lomb, Tampa, FL). The epithelial wound was monitored with fluorescein staining (0.5% sodium fluorescein) and documented using a Micron III imaging system (Phoenix Research Laboratories, Pleasanton, CA, USA). The wound healing process at time 0, 12, 24 and 48 hours were recorded and the animals were euthanized by CO_2_ inhalation and whole globes were collected for histology and FACS studies. Ruby spheres (Meller Optics, Providence, RI, USA) of 0.5 mm diameter were placed near lacrimal puncta on the cornea as a size reference for the fluorescein image analysis to determine the size of the injury. Due to the strong light reflection of the ruby sphere, lower intensity of illuminating light (compared to the intensity used for fluorescein staining of the same viewing field) was used for photography to obtain accurate images of these spheres.

### Analyses of the inflammatory cytokines by Cytometric bead array

Corneas were dissected from enucleated eyes by cutting just central to the limbus to avoid the contribution of resident immune cells, which are abundant in the limbus. The dissected corneas were further cut into approximately 1×1 mm tissue blocks, and then extracted with RIPA buffer. Cytokines (IL-1α, IL-1β, IL-6 and MCP-1) content was measured using BD Cytokine Bead Array Flex Sets (BD Biosciences, San Diego, CA), per manufacturer's protocol.

### Statistical analysis

The results were compared by a two-sample independent Student's t-test to determine if there were statistical differences between WT and L7M1 using the statistical software in Origin version 7.5 (OriginLab, Northampton, MA, USA). The results were considered significant if the probability value was less than 0.05. Data are reported as mean ± SEM.
